# The Therapeutic Implications of Plasticity of the Cancer Stem Cell Phenotype

**DOI:** 10.1371/journal.pone.0014366

**Published:** 2010-12-17

**Authors:** Kevin Leder, Eric C. Holland, Franziska Michor

**Affiliations:** 1 Department of Biostatistics and Computational Biology, Dana-Farber Cancer Institute, and Department of Biostatistics, Harvard School of Public Health, Boston, Massachusetts, United States of America; 2 Cancer Biology and Genetics Program, Department of Neurosurgery, Memorial Sloan-Kettering Cancer Center, New York, New York, United States of America; University of Calgary, Canada

## Abstract

The cancer stem cell hypothesis suggests that tumors contain a small population of cancer cells that have the ability to undergo symmetric self-renewing cell division. In tumors that follow this model, cancer stem cells produce various kinds of specified precursors that divide a limited number of times before terminally differentiating or undergoing apoptosis. As cells within the tumor mature, they become progressively more restricted in the cell types to which they can give rise. However, in some tumor types, the presence of certain extra- or intracellular signals can induce committed cancer progenitors to revert to a multipotential cancer stem cell state. In this paper, we design a novel mathematical model to investigate the dynamics of tumor progression in such situations, and study the implications of a reversible cancer stem cell phenotype for therapeutic interventions. We find that higher levels of dedifferentiation substantially reduce the effectiveness of therapy directed at cancer stem cells by leading to higher rates of resistance. We conclude that plasticity of the cancer stem cell phenotype is an important determinant of the prognosis of tumors. This model represents the first mathematical investigation of this tumor trait and contributes to a quantitative understanding of cancer.

## Introduction

Traditionally, many different cell types within a tumor have been considered to have tumorigenic potential and possess the ability to cause cancers in secondary recipients. By contrast, the cancer stem cell hypothesis suggests that only a small subpopulation of tumor cells has that potential [1]. This hypothesis has been shown consistent with data from such diverse cancer types as chronic and acute myeloid leukemias [2], [3], breast cancer [4], colorectal cancer [5], mesenchymal neoplasms [6], head and neck squamous cell carcinoma [7], and pancreatic cancer [8]. The investigation of cancer stem cells in melanoma, however, has led to controversial findings. Some studies suggested that melanoma cells that are capable of transplanting the disease are exceedingly rare [9] while others, using more severely immunocompromised mice, found that cells with those capabilities are very common within the tumor [10]. Similarly, the frequency of tumor cells positive for stem cell-like markers in breast cancer varies according to the stage and subtype of the tumor [11].

These findings have led to discussions about the applicability of the cancer stem cell hypothesis to all tumor types, and also the ability of xenotransplantation assays to reliably identify cancer stem cells [12], [13]. The differential ability of mouse models to detect cancer stem cells may be explained by a context-dependent phenotype of those cells, as supported by evidence from co-injection experiments of stromal and cancer cells [10]. In these studies, the efficiency of transplantation of putative cancer stem cells was higher when stromal cells were co-injected as compared to injection of cancer stem cells alone. This data suggests that the ability of cells to initiate neoplastic growth may not only depend on the severity of immunodeficiency of assay mice, but also on the microenvironmental context of these cells [14].

The phenotypic plasticity of stem cells has been a topic attracting great interest. Studies of cells in the central nervous system, for instance, have shown that certain extracellular signals can induce oligodendrocyte precursor cells to dedifferentiate into multipotential neural stem cells [15]. These extracellular signals are provided through exposure to fetal calf serum and certain cytokines, including some bone morphogenic proteins, as well as basic fibroblast growth factor (bFGF), and cause many purified oligodendrocyte precursors to revert to a state that resembles that of multipotential neural stem cells [15]. Similarly, a study in which mature astrocytes were exposed to transforming growth factor α (TGFα) demonstrated that a single extracellular factor is sufficient to induce differentiated cells of the central nervous system to regress into a stem-like cell stage [16]. This observed plasticity of normal tissue stem cells has implications for tissue organization in general, and the view of rigid differentiation hierarchies of cells must be revised in light of these findings.

Observations parallel to those observing a dedifferentiation potential of normal cells have also been made with regard to cancer cells. A recent study identified signaling within the perivascular niche as a driving force for tumor cells to acquire stem cell characteristics. In this study, nitric oxide was shown to activate Notch signaling via cGMP and PKG in a subset of glioma cells resulting in acquisition of the side population phenotype and increased neurosphere and tumor formation [17]. These alterations occurred within as little as two hours of treatment and had long-term effects on the phenotype generally associated with stem cell character. This plasticity of tumor stem cells may also apply to liquid tumors, as it was recently shown that leukemia-initiating cells in AML patients harboring mutations in nucleophosmin (NPM) can reside in the CD34+ as well as CD34- fraction [18].

The ability of committed cancer progenitors to dedifferentiate to a stem-like state has important implications for the dynamics of tumor progression and the response to therapy. In this paper, we design a novel mathematical model to quantify the effects of the dedifferentiation rate on disease outcome. As all mathematical modeling approaches, our framework represents an abstraction of the biological system and as such should be considered as a toy model to investigate several characteristics of the system. This work is part of a growing literature describing mathematical investigations of cancer stem cells [19], [20], [21], [22], [23], [24].

## Methods

**Figure 1:**
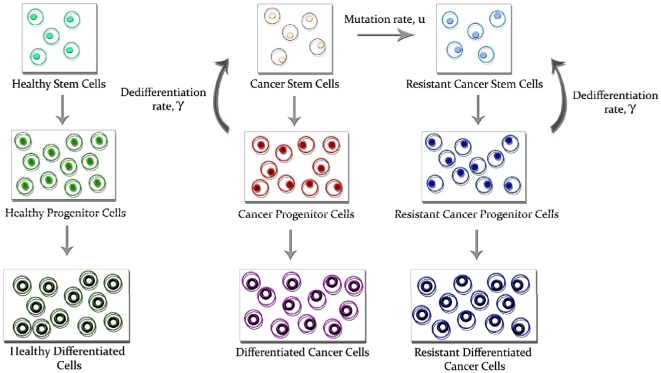
Schematic representation of the mathematical model. The mathematical model considers three levels of the differentiation hierarchy of cells: stem cells, progenitors and differentiated cells. These cell types are present in the system as healthy cells (left), drug-sensitive cancer cells (middle) and drug-resistant cancer cells (right). Stem cells give rise to progenitors which in turn give rise to differentiated cells. Additionally, cancer progenitors may have the ability to dedifferentiate to stem cells. The rate of dedifferentiation is denoted by *γ*. Drug-sensitive cancer stem cells produce drug-resistant cancer stem cells at rate *u* per cell division.

vWe designed a simple mathematical model to investigate the dynamics of different cell populations during tumor progression and treatment. The model considers three differentiation stages for both the healthy and the cancer cell differentiation hierarchies. Stem cells reside at the top of the hierarchy and give rise to progenitor cells, which in turn give rise to differentiated cells (Fig. 1). Denote the abundances of healthy stem, progenitor, and differentiated cells by *x*_0_, *x*_1_, and *x*_2_, respectively, and the abundances of the corresponding cancer cell types by *y*_0_, *y*_1_, and *y*_2_. Healthy stem cells proliferate at rate *r_x_* and die at rate *d*_0_, and give rise to healthy progenitors at rate *a_x_* per day; the rate *a_x_* also includes the possibility of limited expansion in the progenitor compartment via symmetric self-renewing progenitor cell divisions. Progenitors die at rate *d*_1_ and give rise to healthy differentiated cells at rate *b_x_* per day; the latter cells die at rate *d_2_*. Similarly, cancer stem cells proliferate at rate *r_y_* and give rise to cancer progenitors at rate *a_y_*, which in turn give rise to differentiated cancer cells at rate *b_y_* per day. Again, the rate *b_y_* includes the possibility of differentiated cell replication. The death rates per day of the cancer cell types are denoted by *c*_0_, *c*_1_, and *c*_2_. In the simplest form of our model, we consider these parameters to be constant unless external factors – such as the administration of treatment – are applied to the system. However, the model can easily be extended to include more complex scenarios such as variability in the microenvironment, involvement of the immune system, and interactions between cancer and stromal cells. Such situations may be described by considering a distribution of parameters from which the values are selected. In the absence of estimates for the parameters and their distributions, however, we chose to analyze the model in its simpler form of constant parameter values.

In addition to their ability to produce differentiated cancer cells, cancer progenitors may regress to a stem-like state via genetic, epigenetic, or other mechanisms [17]. The rate at which cancer progenitors dedifferentiate per day is denoted by *γ*. This rate may be a function of the microenvironmental conditions of these cells and can also vary over time as tumors become more aggressive. There may also be a similar propensity of healthy progenitor cells to regress to a stem cell like state [15], [16]; a rate of dedifferentiation of healthy progenitors can be included in the model but is neglected for purposes of clarity. We consider the production of stem cells to be limited by the density of both healthy and cancer stem cells; this modeling assumption is made because stem cell numbers are limited by the availability of extracellular resources as well as spatial constraints within the tissue, and therefore the production of stem cells is constrained by those resources and spatial considerations regardless of whether they are produced by symmetric division or dedifferentiation. The density dependence is achieved by the functions 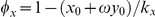
and 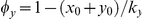 for these two cell types. The terms *k_x_* and *k_y_* represent the carrying capacity that the healthy and cancer stem cells may expand to, and the term *ω* represents the increased crowding that cancer cells can tolerate. It is for this density dependence effect that we include the healthy differentiation hierarchy in our system. Note that the parameters *k_x_* and *k_y_* can be scaled to describe situations with extensive competition between cells (large extent of density dependence) as well as situations with little competition.

Then the basic mathematical model is given by 
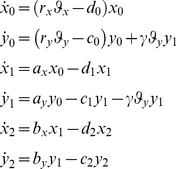
(1)


For shorthand we will write 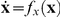 and 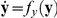 instead of equation (1). In order to study the dynamics of these cells in response to therapy, we denote the rates of production of cancer cells during treatment by 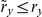, 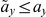, and 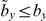. We may also investigate an additional or alternative effect of therapy on the death rates of cancer cells, leading to increased rates during treatment: 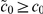, 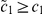, and 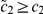. Note that we do not allow the terminally differentiated cells to dedifferentiate to give rise to progenitors; this modeling assumption is made since in most tumor types, terminally differentiated cancer cells are post-mitotic and therefore incapable of dedifferentiating.

The model outlined above considers the dynamics of treatment response without the possibility of acquired resistance. Even drugs that elicit a dramatic initial response often fail later on due to the emergence of resistance mutations which render the drug ineffective. Two prominent examples are the point mutations in BCR-ABL and EGFR that confer resistance against the small molecule inhibitors imatinib/dasatinib and erlotinib/gefitinib [25], [26]. In the context of our model, the first cell carrying a resistance mutation can only initiate a long-lived clone if it is a stem cell, or alternatively a progenitor that dedifferentiates to a stem-like state. Denote the rate at which a resistance mutation arises per cell division by *u*. The probability of resistance depends on the total number of stem cell divisions and a fraction of progenitor cell divisions, given by
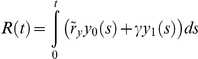



Note that a resistant cell can arise during a division of a sensitive cancer stem cell or during a dedifferentiation event of a sensitive cancer progenitor cell. Then the probability that at least one resistant cell that will persist in the population has arisen by time *t* is given by 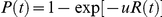.

Furthermore, the basic mathematical model as given by equation (1) can be expended to include a differentiation hierarchy of drug-resistant cancer cells. Denote the abundance of resistant stem, progenitor and differentiated cancer cells by *z*_0_, *z*_1_ and *z*_2_, respectively. Then the dynamics of the system including resistant cells is given by 
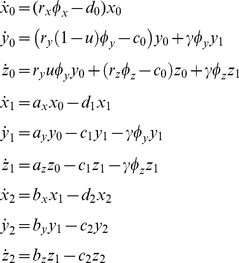
(2)


Here the growth, death and differentiation rates of resistant cancer cells are denoted by the parameters *r_y_*, *a_z_, b_z_,* and *c_0_, c_1_,* and *c_2_*, respectively.

## Results

**Figure 2:**
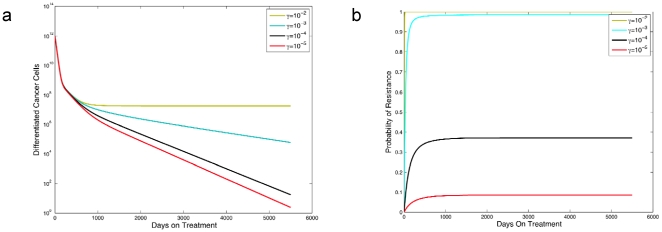
The effect of dedifferentiation on the abundance of differentiated cancer cells and the probability of resistance. In panel **a**, we show the abundance of differentiated cancer cells over time since the initiation of therapy. In panel **b**, we plot the probability of resistance versus time. Growth rates during treatment are 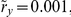 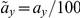 and 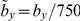, and death rates are 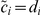 for *i* = 0,1,2. Other parameters are *r_x_* = 0.005, *r_y_* = 0.008, *d_0_* = 0.004, *d_1_* = 0.008, *d_2_* = 0.05, *a_x_* = 100*d_1_*, b*_x_* = 100*d_2_*, *a_y_* = 2*a_x_*, *b_y_* = 2*b_x_*, *k_x_* = 1.2×10^6^, *k_y_* = 6×10^7^, *u* = 5×10^−9^, and ω = 0.1. The initial condition for the panels is found by simulating system (1) using the pretreatment parameter values and the initial condition *x*_0_(0)  = 10^6^, *x*_1_(0)  = 10^8^, *x*_2_(0)  = 10^10^, *y*_0_(0)  = 1, and *y*_1_(0)  = *y*_2_(0)  = 0. We simulate this system until detection time *T*, i.e., when *y*_2_(*T*) ≥10^12^, and then simulate the treatment phase by running system (1) with the initial conditions *x*_0_(*T*), *x*_1_(*T*), …, *y*_2_(*T*) and the treatment parameter values.

Let us first discuss the effects of the dedifferentiation parameter on the dynamics of treatment response. Figure 2a shows the number of differentiated cancer cells as a function of time after the initiation of therapy. Note that the dedifferentiation parameter *γ* has a strong affect on the tumor's response to treatment; in particular, a larger value of *γ* corresponds to a poorer response to treatment. In Figure 2b, we investigate the effect of the dedifferentiation rate, *γ*, on the probability of resistance, *R*(*t*), finding that larger values of *γ* lead to a substantially higher risk of developing resistance.

### The dynamics of treatment response without resistance mutations

Let us now consider specific examples for the treatment response of a tumor for a fixed level of the dedifferentiation parameter, *γ*. In the following we investigate the effects of a variety of hypothetical drugs that target different cells within the differentiation hierarchy. Note that these scenarios describe idealized treatments which exert the specified effects onto cancer cells; these scenarios serve as examples of the dynamics of the system during drug treatment. Table 1 provides a summary of the four treatment strategies considered.

**Table 1:** The effect of different hypothetical treatment strategies on cancer cell populations.

	Stem Cells	Progenitor Cells	Differentiated Cells
Treatment 1	−	+	+
Treatment 2	+	+	+
Treatment 3	+	+	+
Treatment 4	+	−	−

Sensitivity to treatment is denoted by ‘+’ and insensitivity by ‘−’.

**Figure 3:**
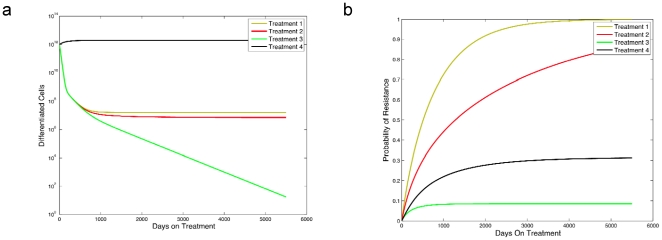
The effect of different treatment strategies on the abundance of differentiated cancer cells and the probability of resistance. The figure shows the abundance of differentiated cancer cells, *y_2_*, over time since initiation of therapy in panel **a** and the probability of resistance, *P*(*t*), as a function of time in panel **b**. We display four different treatment types that affect the cancer cell populations differentially. *Treatment 1* represents a drug that affects only the production of cancer progenitor and differentiated cells, 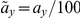 and 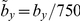. *Treatment 2* is a drug affecting all cancer cell types while not inhibiting cancer stem cells by a substantial amount, 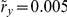 while 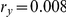, and 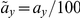 and 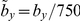. *Treatment 3* represents a drug that affects all cancer cell types and has a substantial effect on stem cells, 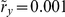, 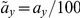 and 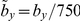. *Treatment 4* is a drug that decreases only the growth rate of cancer stem cells, 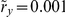. The pre-treatment parameters are identical to those in Figure 2, and in both panels we set 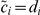 for *i* = 0,1,2.

First, let us investigate a hypothetical drug that reduces the production rate of both progenitor and differentiated cells. *Treatment 1* in Figure 3 provides a numerical example for such a situation. The panels of the figure show the abundance of differentiated cancer cells over time in response to treatment (Fig. 3a) as well as the probability of resistance emerging during treatment (Fig. 3b). Note that this type of treatment leads to a reduction in the number of cancer progenitors and differentiated cells, but is incapable of depleting cancer stem cells. Such interventions might reduce symptoms for a limited time by debulking the tumor. However, the persistence of cancer stem cells in this scenario prevents tumor eradication and permits the evolution of acquired resistance, which renders the response to treatment short-lived.

In contrast to the scenario above, a drug may inhibit the production of all cancer cell types but still fail to completely eradicate the cancer cell population. To illustrate this point, consider a drug that inhibits all three cancer cell types. A drug that elicits this response is shown as *Treatment 2* in Figure 3. The performance of a drug with these properties is a slight improvement over the example considered previously; however, the probability that acquired resistance will evolve is still substantial since the drug cannot deplete the cancer stem cell population. This type of drug allows a stable population of cancer stem cells to remain and continuously repopulate the progenitor and differentiated cells.

Let us now consider a drug that reduces the growth rate of all cancer cell types to a larger extent. An example of this type of therapy is shown as *Treatment 3* in Figure 3. A drug eliciting these effects is capable of eradicating the disease. Furthermore, the probability of resistance is small since the cancer stem cell population is driven to extinction and thus, fewer chances for the emergence of a resistance mutation to arise. Once treatment has eradicated cancer stem cells, cancer progenitor and differentiated cells equally go extinct since the latter cell types do not have (sufficient) self-renewing abilities.

Lastly, let us consider a drug that inhibits cancer stem cells only. In this setting, the rate of depletion of the total cancer cell population may be too slow to be considered effective; an example is shown as *Treatment 4* in Figure 3. The advantage of such a drug is that the chance of drug-resistant tumors is diminished since there are so few stem cells. However, the tumor burden remains at a relatively high level for a prolonged period of time because differentiated cancer cells are unaffected by the drug.

These four treatment strategies represent idealized therapies; however, their study leads to insights into how heterogeneous tumor cell populations respond to treatments that affect particular types of cells, and suggests the most desirable subpopulation to target.

### The dynamics of treatment response with resistance

**Figure 4:**
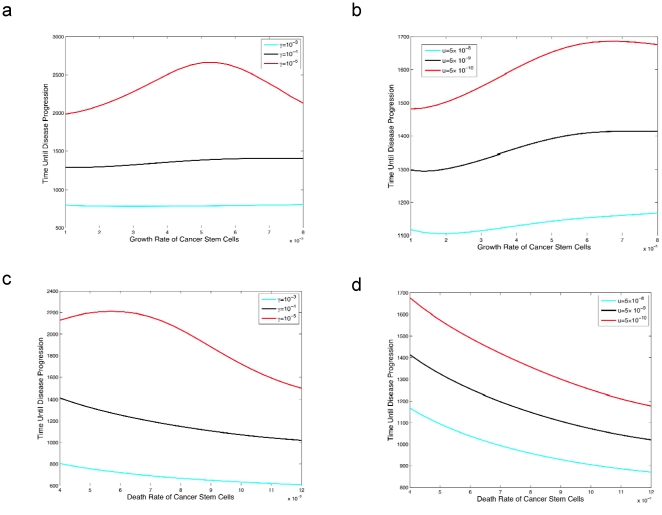
The effect of different cancer stem cell treatment strategies on the time until disease progression. The figure shows the time until the disease burden increases despite continuous therapy versus the birth rate (panels **a** and **b**) and death rate (panels **c** and **d**) of cancer stem cells during therapy. The pre-treatment growth parameters are identical to those in Figure 2, and also 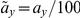 and 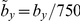, lastly we set 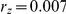 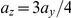 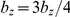. In panel **a,** we set 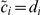 for *i* = 0,1,2, and *u* = 5×10^−9^. The parameter 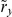 varies along the x-axis and we consider three different values of *γ*. In panel **b**, we set 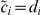 for *i* = 0,1,2, and *γ* = 10^−4^. The parameter 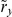 varies along the x-axis and we consider three different values of *u*. In panel **c**, we set 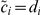 for *i* = 1,2, *u* = 10^−7^, 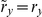, vary 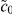 along the x-axis and consider three different values of *γ*. In panel **d**, we set 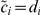 for *i* = 1,2, *γ* = 10^−4^, 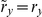, vary 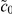 along the x-axis and consider three different values of *u*.

Instead of considering the proportion of patients that develop resistance, it is also useful to investigate the expected number of resistant cells present within a patient for a given mutation rate, *u*. In the context of our mathematical model, drug-resistant cancer stem cells initiate their own cellular differentiation hierarchy (Fig. 1 and equation (2)). With this extension of the mathematical model, we can investigate an additional aspect of treatment: the propensity of a drug to delay progression due to resistance, i.e. the onset of a resistance-driven rebound of the cancer cell population. Figure 4 shows how drugs that target various parameters of the cancer cell differentiation hierarchy can have vastly different effects on the duration of successful treatment before the onset of resistance.

When comparing drugs that affect the birth and death rates of cancer stem cells, drugs that target the production of cancer stem cells lead to a longer time during which treatment is effective and before resistance emerges. This effect can be seen by comparing panels a and b with panels c and d of Figure 4, and results from the fact that a reduction in the number of cancer stem cell divisions leads to fewer opportunities per unit time for resistant cells to arise. Figure 4 also shows that dedifferentiation can have a very strong effect on the time until disease progression. In particular, a change in the order of magnitude of the dedifferentiation rate has approximately twice the effect as compared to a change in magnitude of the mutation rate. Lastly, note that an increased net growth rate of stem cells delays the rebound of the tumor population. This fact is due to the maintenance of the stem cell population near its carrying capacity, which prevents resistant cell populations from arising since we consider density-dependent growth dynamics.

**Figure 5:**
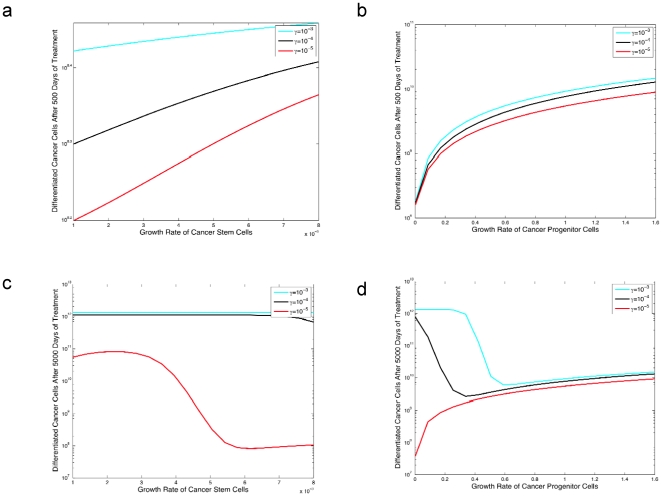
The effect of different cancer treatment strategies on the number of differentiated cancer cells in the presence of resistance. Panels **a** and **b** display the tumor cell population after 500 days of treatment for two different types of treatment. In panel **a** we consider a treatment that can target all types of cells, and in panel **b** we consider a treatment that only targets progenitor and differentiated cells. Panels **c** and **d** display the tumor cell population after 5000 days of treatment for two different types of treatment. In panel **c** we consider a treatment that can target all types of cells, and in panel **d** we consider a treatment that only targets progenitor and differentiated cells. The pre-treatment growth parameters are identical to those in Figure 2 and the growth rate of the resistant cells is identical to that in Figure 4. In all four panels we set *u* = 5×10^−9^ and we set 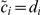 for *i* = 0,1,2. In panels **a** and **c** we set 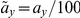 and 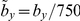, and vary 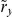 (i.e., the drug effect on cancer stem cells) along the horizontal axis. In panels **b** and **d**, we set 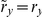 and 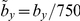, and vary 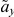 (i.e., the drug effect on cancer progenitors) along the horizontal axis. In panels a and b, the vertical axis corresponds to the number of differentiated cancer cells after 500 days of treatment, including resistant and sensitive cancer cells. In panels **c** and **d**, the vertical axis corresponds to the number of differentiated cancer cells after 5000 days of treatment, including resistant and sensitive cancer cells.

Let us now compare the efficacy of different treatment protocols while also taking into consideration the possibility of resistance (Fig. 5). We investigate two types of treatment: a drug that causes a decline in the growth rates of all cancer cell types (Fig. 5 a and c), and a drug that only inhibits progenitor and differentiated cells (Fig. 5b and d). When comparing the effectiveness of these two drugs over short time spans (panels a and b), we find that a drug that inhibits proliferation for all cell types is preferable as compared to the other type of treatment – i.e., the total cell number is significantly lower in the former case. However, over longer time spans (panels c and d) the drug that only inhibits progenitor and differentiated cells is more effective at preventing the emergence of resistance. This effect results from the density dependent growth of cancer stem cells; since this drug does not inhibit the stem cell population, resistant stem cells never become established due to the density constraint mechanism. Any resistant stem cells that arise during administration of this treatment will have suppressed growth since the stem cell population has already reached its maximum population size. Note that the short time span (panels a and b) refers to 500 days after the initiation of therapy, while the long time span (panels c and d) refers to 5000 days since the start of treatment. The drug shown in panels a and b does significantly decrease the population of stem cells, and therefore any resistant stem cell that arises will not be inhibited by the density constraint mechanism and be able to establish a resistant clone. Hence in the short term, it is preferable to inhibit cancer stem cells (panels a and b), while during longer periods of time (panels c and d), this strategy may backfire because it allows the resistant tumor stem cells to grow.

**Figure 6:**
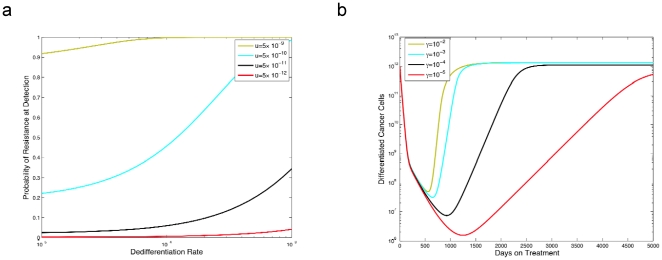
The relationship between dedifferentiation rate and pre-existing resistance. Panel **a** considers the probability of pre-existing resistance versus the dedifferentiation rate *γ* for several mutation rates. We use the same pre-treatment growth rates as in **Figure 2** and the same growth rates for the resistant cells as in **Figure 4**, and evolve the system until the tumor population hits size 10^12^ and then evaluate the probability of resistance at that time. Panel **b** plots the response of a tumor population to a drug, assuming that pre-existing resistant population of cells is present at beginning of treatment. The sensitive cells have the same growth rate as in **Figure 2**, and the resistant cell have the same growth rates as in **Figure 4** **.**

A reduction of the dedifferentiation rate has a beneficial effect regardless of the cell type that the drug targets (Fig. 5). However, note in Figure 6d that the sensitivity of the system to the dedifferentiation parameter is decreased with increasing progenitor birth rate, *a_y_*. An increase in the production of progenitor cells leads to a larger number of those cells, and thus a decrease in the dedifferentiation rate will need to be enhanced in order to have a substantial effect on the stem cell population.

### Dedifferentiation increases the risk of pre-existing resistance

In many cases of treatment failure due to the evolution of acquired resistance, resistant cells are present at the time of diagnosis. Let us thus discuss such pre-existent resistance and its effects on the prognosis of cancer patients (Fig. 6). First we study the probability of developing resistance prior to detection. Fig. 6a shows that as the dedifferentiation rate increases, the probability of pre-existing resistance also increases. This effect arises because there are more opportunities for resistant cancer stem cells to arise if an increasing fraction of progenitor cells dedifferentiate to become cancer stem cells, since we consider the contribution of dedifferentiating progenitor cells to the total risk of resistance in the stem cell pool. Fig. 6b displays the number of tumor cells as a function of time after the initiation of therapy under the assumption that a small number of resistant cells is present at the time of diagnosis. Note that the time until the resistant cells cause a disease rebound is strongly dependent on the dedifferentiation rate – the larger this rate becomes, the more rapidly the cancer cell population rebounds due to the expansion of resistant cells. This finding is consistent with the results shown in Figure 4.

### The success of pulsatile therapy depends on the extent of dedifferentiation

Anti-cancer therapy is often administered in treatment pulses to limit the toxicity of these agents. The advantage of treatment pulses is that higher drug concentrations can be reached using such a strategy as compared to the continuous dosing regimen. The disadvantage to pulsatile therapy, however, is that during treatment breaks, the cancer cell population expands exponentially and leads to rebounds as well as an increased risk of acquired resistance. So far, the effects of dedifferentiation of progenitor cells to a stem cell-like state have not been investigated with respect to pulsed treatment strategies. Our mathematical model is useful for evaluating the impact of pulsed therapy with regard to recovery of the cancer stem cell population by dedifferentiation of progenitors.

**Figure 7:**
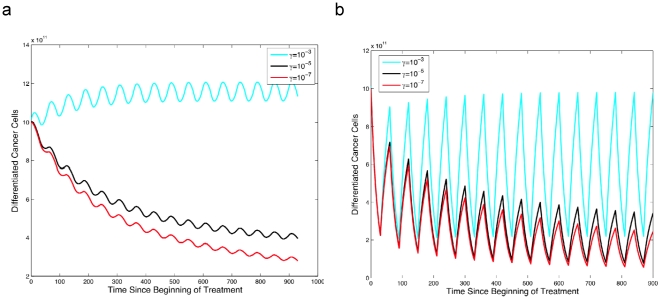
The effect of the dedifferentiation rate on differentiated cancer cells during pulsatile therapy. The figure shows the dynamics of differentiated cancer cells in response to a treatment strategy in which the drug is administered for 30 days, followed by a treatment holiday of 30 days during each pulse. Panel **a** shows the effects of a drug which inhibits cancer stem cell proliferation and their differentiation to progenitors, while panel **b** demonstrates the effects of a drug which additionally inhibits the production of differentiated cancer cells from progenitors. Parameters are 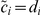 for *i* = 0,1,2, 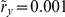, 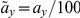, and 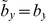 in (**a**) and 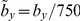 in (**b**). For both panels, the off-treatment parameters are identical to those in Figure 2.

Figure 7 shows that higher levels of dedifferentiation, i.e. a larger rate *γ*, lead to larger rebounds of the cancer cell population during treatment breaks as well as lower levels of cancer cell depletion during treatment. Hence the possibility of dedifferentiation of cancer progenitors renders otherwise successful therapy less effective, to the point of being unsuccessful if the capacity of progenitors for dedifferentiation is sufficiently large. Fig. 7a demonstrates the effects of treatment with a drug that only inhibits the production of cancer stem and progenitor cells. This scenario leads to a drastic difference between situations with different levels of the dedifferentiation parameter *γ*. Figure 7b shows the treatment response to a drug that additionally inhibits the production of differentiated cancer cells. In this situation, the difference between varying values of *γ* is not as pronounced since the drug has a substantial effect on differentiated cancer cells.

## Discussion

During normal development, differentiation from stem cells to final products is unidirectional. Some data suggest that oncogenic mutations lead to loss of the ability for cells to maintain their differentiated state. In the case of tumor suppressors, their normal function may therefore be to maintain the unidirectional flow of differentiation during development. Their inactivation or alteration of certain signaling pathways may result in the loss of unidirectionality of this process. Dedifferentiation does not necessarily refer to a scenario in which every cell reverses its differentiation phenotype. Instead, a small fraction of committed progenitor cells may acquire stem cell-like characteristics in response to genetic or environmental changes, and this small cell number may lead to qualitatively different dynamics. We refer to the fraction of cells that dedifferentiate per time unit as gamma. In this paper, we determine the effects of various values of gamma on the response of tumors to therapy, which specifically targets either stem cells or non-stem cells. We have studied four hypothetical treatment strategies (see Table 1 and the results section) and have also evaluated the effects of dedifferentiation on the risk of pre-existing therapy and the success of pulsatile treatment.

We chose to formulate a simple mathematical framework that only incorporates the essential considerations of cancer stem, progenitor and differentiated cells. While our mathematical model can easily be extended to describe more complicated scenarios such as interactions of cancer cells with the stroma and immune system, and the generation of tumor cell heterogeneity through other avenues such as clonal diversification, we have concentrated on the analysis of the basic model during different treatment options. This model will be extended in future work to consider more complex situations in cancer.

The results obtained from this modeling study indicate that the response of tumors capable of dedifferentiating is qualitatively different from a scenario in which treatment cannot completely eradicate the bulk of tumor cells and the remaining cells lead to a rebound post-therapy. In the latter case, the remaining cells likely are sensitive to being re-challenged with treatment and therefore, pulsed therapy has the potential to eradicate the disease. In a scenario including the potential of dedifferentiation of cells, pulsed therapy targeting stem cells is incapable of curing the disease, since the cancer stem cell pool is continuously repopulated by progenitor cells during treatment breaks. Therefore, the consideration of a dedifferentiation potential of cancer cells is important for an accurate understanding of anti-cancer therapy.

There has been significant discussion of the effects of tumor stem cells that are insensitive to anti-cancer therapy. Standard therapy inhibits proliferating cells of the tumor bulk but the tumors recur from drug-insensitive stem cells. Because of this, it has been suggested that a strategy targeting cancer stem cells is required for curative therapy. However, if non-stem cells can acquire stem cell properties with a sufficiently high probability but still much lower than the differentiation rate, then a stem cell-specific treatment strategy will be futile. The results of our mathematical modeling studies described in this paper suggest that higher levels of dedifferentiation substantially reduce the effectiveness of therapy directed at cancer stem cells. During pulsed treatment strategies, the possibility of dedifferentiation leads to higher rebounds of the cancer cell population during treatment breaks as well as lower levels of cancer cell reduction during treatment pulses. In addition, we see that increasing the level of dedifferentiation significantly increases the number of stem cell replications and therefore increases the probability of acquiring a resistance mutation in a stem cell. In summary, plasticity of the cancer stem cell phenotype is an important determinant of the effectiveness of therapy, and its possibility cannot be neglected to gain an accurate understanding of the treatment response of human tumors.

## References

[1] Gupta PB, Chaffer CL, Weinberg RA (2009). Cancer stem cells: mirage or reality?. Nature Medicine.

[2] Wang JCY, Lapidot T, Cashman JD, Doedens M, Addy L (1998). High level engraftment of NOD/SCID mice by primitive normal and leukemic hematopoietic cells from patients with chronic myeloid leukemia in chronic phase.. Blood.

[3] Hope KJ, Jin LQ, Dick JE (2004). Acute myeloid leukemia originates from a hierarchy of leukemic stem cell classes that differ in self-renewal capacity.. Nature Immunology.

[4] Al-Hajj M, Wicha MS, Benito-Hernandez A, Morrison SJ, Clarke MF (2003). Prospective identification of tumorigenic breast cancer cells.. Proceedings of the National Academy of Sciences of the United States of America.

[5] Ricci-Vitiani L, Lombardi DG, Pilozzi E, Biffoni M, Todaro M (2007). Identification and expansion of human colon-cancer-initiating cells.. Nature.

[6] Wu CL, Wei QX, Utomo V, Nadesan P, Whetstone H (2007). Side population cells isolated from mesenchymal neoplasms have tumor initiating potential.. Cancer Research.

[7] Prince ME, Sivanandan R, Kaczorowski A, Wolf GT, Kaplan MJ (2007). Identification of a subpopulation of cells with cancer stem cell properties in head and neck squamous cell carcinoma.. Proceedings of the National Academy of Sciences of the United States of America.

[8] Li CW, Heidt DG, Dalerba P, Burant CF, Zhang LJ (2007). Identification of pancreatic cancer stem cells.. Cancer Research.

[9] Schatton T, Murphy GF, Frank NY, Yamaura K, Waaga-Gasser AM (2008). Identification of cells initiating human melanomas.. Nature.

[10] Quintana E, Shackleton M, Sabel MS, Fullen DR, Johnson TM (2008). Efficient tumour formation by single human melanoma cells.. Nature.

[11] Park SY, Lee HE, Li H, Shipitsin M, Gelman R (2010). Heterogeneity for stem cell-related markers according to tumor subtype and histologic stage in breast cancer.. Clin Cancer Res.

[12] Kelly PN, Dakic A, Adams JM, Nutt SL, Strasser A (2007). Tumor growth need not be driven by rare cancer stem cells.. Science.

[13] Visvader JE, Lindeman GJ (2008). Cancer stem cells in solid tumours: accumulating evidence and unresolved questions.. Nature Reviews Cancer.

[14] Pietras K, O A (2010). Hallmarks of cancer: Interactions with the tumor stroma.. Exp Cell Res.

[15] Kondo T, Raff M (2000). Oligodendrocyte precursor cells reprogrammed to become multipotential CNS stem cells.. Science.

[16] Sharif A, Legendre P, Prévot V, Allet C, Romao L (2007). Transforming growth factor alpha promotes sequential conversion of mature astrocytes into neural progenitors and stem cells.. Oncogene.

[17] Charles N, Ozawa T, Squatrito M, Bleau AM, Brennan CW (2010). Perivascular Nitric Oxide Activates Notch Signaling and Promotes Stem-like Character in PDGF-Induced Glioma Cells.. Cell Stem Cell.

[18] Taussig DC, Vargaftig J, Miraki-Moud F, Griessinger E, Sharrock K (2010). Leukemia-initiating cells from some acute myeloid leukemia patients with mutated nucleophosmin reside in the CD34- fraction.. Blood.

[19] Michor F (2008). Mathematical models of cancer stem cells.. Journal of Clinical Oncology.

[20] Ganguly R, Puri IK (2006). Mathematical model for the cancer stem cell hypothesis.. Cell Proliferation.

[21] Dingli D, Traulsen A, Pacheco JM (2007). Stochastic dynamics of hematopoietic tumor stem cells.. Cell Cycle.

[22] Turner C, Stinchcombe AR, Kohandel M, Singh S, Sivaloganathan S (2009). Characterization of brain cancer stem cells: a mathematical approach.. Cell Proliferation.

[23] Haeno H, Levine RL, Gilliland DG, Michor F (2009). A progenitor cell origin of myeloid malignancies.. Proceedings of the National Academy of Sciences of the United States of America.

[24] Sottoriva A, Verhoeff JJ, Borovski T, McWeeney SK, Naumov L (2010). Cancer stem cell tumor model reveals invasive morphology and increased phenotypical heterogeneity.. Cancer Res.

[25] Gorre ME, Mohammed M, Ellwood K, Hsu N, Paquette R (2001). Clinical resistance to STI-571 cancer therapy caused by BCR-ABL gene mutation or amplification.. Science.

[26] Pao W, Miller VA, Politi KA, Riely GJ, Somwar R (2005). Acquired resistance of lung adenocarcinomas to gefitinib or erlotinib is associated with a second mutation in the EGFR kinase domain.. Plos Medicine.

